# Grape-Derived Polysaccharide Extracts Rich in Rhamnogalacturonans-II as Potential Modulators of White Wine Flavor Compounds

**DOI:** 10.3390/molecules28186477

**Published:** 2023-09-06

**Authors:** Diego Canalejo, Leticia Martínez-Lapuente, Belén Ayestarán, Silvia Pérez-Magariño, Thierry Doco, Zenaida Guadalupe

**Affiliations:** 1 Instituto de Ciencias de la Vid y del Vino (Universidad de la Rioja, Gobierno de La Rioja y CSIC), Ctra. De Burgos Km 6, 26007 Logroño, Spain; diego.canalejo@outlook.es (D.C.); leticia.martinez@unirioja.es (L.M.-L.); belen.ayestaran@unirioja.es (B.A.); 2 Instituto Tecnológico Agrario de Castilla y León, Consejería de Agricultura y Ganadería, Ctra. Burgos Km 119, 47071 Valladolid, Spain; permagsi@itacyl.es; 3 SPO, INRAE, Institut Agro, Univ Montpellier, 2 Place Pierre Viala, F-34060 Montpellier, France; thierry.doco@inrae.fr

**Keywords:** rhamnogalacturonans type II (RG-II), mannoproteins (MP), polysaccharides rich in arabinose and galactose (PRAG), winemaking by-products, volatiles, phenols sensory quality

## Abstract

Many authors have investigated the role of mannoproteins on wine quality, but very few have analyzed the use of grape-derived polysaccharides as they are not commercially available. In this study, purified grape-derived polysaccharides from red wine (WPP) and winemaking by-products (DWRP: Distilled Washing Residues Polysaccharides) were used as potential fining agents to modulate white wine flavor. Phenolics and volatile compounds were analyzed in the control and wines treated with WPP, DWRP, and commercial mannoproteins (CMs) after one and twelve months of bottling, and a sensory analysis was conducted. WPP and DWRP, rich in rhamnogalacturonans-II, showed themselves to be good modulators of wine aroma and astringency. Improvement in wine aroma was related to an increase in all volatile families expect higher alcohols and volatile acids. The modulation of astringency and bitterness was related to a reduction in the proanthocyanidin content and its mean degree of polymerization. Extracts with polysaccharides with higher protein contents presented a higher retention of volatile compounds, and DWRP extract had more positive effects on the overall aroma. Our novel results present the possibility of obtaining valuable polysaccharides from distilled washing residues of wine pomaces, which could promote its valorization as a by-product. This is the first time the potential use of this by-product has been described.

## 1. Introduction

One of the key factors that greatly affect the final product in winemaking is the use of fining agents. Fining agents are widely used in the wine industry to stabilize, clarify, and modify the wine’s sensory properties. The fining agent reacts with wine components either chemically or physically to remove unwanted particles and impurities. These agents can improve the color, flavor, aroma, and texture of the wine since they bind or adsorb particulate matter [1]. However, the choice of the fining agent and the amount used can have a significant effect on the wine’s sensory properties and overall quality. Therefore, it is important to know the effects of the different fining agents and optimize their use to produce high-quality wines that meet consumer expectations. This operation frequently uses proteins of animal origin such as gelatin, egg albumin, casein, and fish proteins. Nevertheless, the allergenic potential of animal proteins has supposed an increased interest in the development of alternative solutions. These may include the addition of proteins obtained from plants, such as those derived from potatoes, cereals, legumes, or grape seeds. Additionally, non-proteinaceous plant-based substances, such as cell wall polysaccharides and pomace materials, have also emerged as potential alternatives to animal proteins for wine fining treatments [2].

From these cell wall polysaccharides, mannoproteins (MPs) are preferred over other fining agents due to their commercial availability and their ability to effectively remove unwanted particles without significantly affecting the sensory properties of the wines. MPs are a group of glycoproteins naturally present in wine which arise from the yeast cell walls [3]. MPs have proved to improve wine sensory properties, such as aroma [4,5,6,7] and mouthfeel [8,9], enhancing the perception of body and texture in wine, as well as increasing color [8] and protein and tartrate stability [10]. These products are specially recommended in white wines to protect aroma and enhance complexity, increasing the perception of fruity aromas and their intensity and refreshing the aromatic potential of already oxidized wines [7]. 

Although grape and wine polysaccharides (PSs) also affect wine sensory quality and chemical composition [11,12,13,14,15,16], they are not commercially available and there is very little research evaluating their potential uses in real wine samples. An arabinogalactan protein (AGP) isolated from a *Carignan noir* red wine enhanced the volatility of some volatile compounds, while rhamnogalacturonan type II (RG-II) polysaccharides decreased the volatility of some esters in model systems [13]. Arabinogalactan (AG) has also been reported to interact with other macromolecules in the wine matrix, such as tannins, forming complexes which produce extra hydrophobic regions, resulting in the retention of volatile compounds [15]. Moreover, AG and RG-II can modulate tannin self-aggregation, having a direct impact on the wine mouthfeel, body, and astringency perception [12,14]. Moreover, a recent study of our workgroup used the polysaccharides obtained from grape pomace and must during wine deposit storage and observed an improvement in some wine properties, such as volatile and polysaccharide composition [16]. Considering the different results obtained in some studies [17], it is essential to investigate the effects of grape polysaccharide on real wine samples instead of model systems. 

Grape polysaccharides arise from the cell walls of grape berries and include non-pectic polysaccharides like celluloses and hemicelluloses, pectic polysaccharides like rhamnogalacturonans type I and II (RG-I and RG-II), homogalacturonans (HGs), and polysaccharides rich in arabinose and galactose (PRAG), which include arabinans and arabinogalactans (AGs), and arabinogalactan proteins (AGPs). RG-II dimer is a complex pectic polysaccharide of the cell walls of all higher plants [18]. It shows the molecular weights of 10 kDa [6] and a complex structure of glycosyl residues [17]. Despite its widespread presence in higher plants, RG-II molecules are difficult to extract as they involve several extraction steps and, therefore, are not commercially available. PRAG are glycoproteins composed of proteins (~10%) and polysaccharides rich in arabinose and galactose (~90%) [19]. HGs consist of α-1,4 D-galacturonic acid residues, which can be methyl-esterified or acetylated; however, during maturation, the de-esterification process allows calcium cations to create crosslinking bridges with other HGs [20,21,22]. Both RG-II and PRAG are present in high amounts in wines, with contents up to 300 and 600 mg L^−1^, respectively. Although HGs can be identified in musts after grape crushing, few HGs are present in the final wines as they might undergo fragmentation by grape or yeast poly-galacturonases.

In previous research, our group developed extraction methods to obtain valuable active polysaccharides from grape pomaces [23] and characterized polysaccharide extracts obtained from grape pomace, musts, wines, lees, and other by-products [24]. This paper aims to evaluate the potential use of highly purified polysaccharide extracts rich in RG-II as fining agents to modulate the flavor composition and sensory properties of white wines. It describes the use of two PS extracts obtained from wine and pomace by-products and compares their effects with a commercial mannoprotein product recommended for this purpose. 

## 2. Results and Discussion

### 2.1. Oenological Parameters

Average ethanol degree was 12.3 ± 0.11%; titratable acidity was 6.11 ± 0.09 g L^−1^ of tartaric acid; volatile acidity was 0.22 ± 0.05 g L^−1^ of acetic acid; malic acid was 1.59 ± 0,06 g L^−1^; pH was 3.43 ± 0.10; free SO_2_ was 29.6 ± 1.2 mg L^−1^; total SO_2_ was 102.3 ± 1.8 mg L^−1^; and the absorbance at 420 nm was 0.13 ± 0.02. The oenological parameters were like those described for this variety [25]. 

### 2.2. Volatile Composition of Viura Wines Treated with PS extracts and CM

Table 1 shows the concentration of individual volatile compounds and volatile families of Viura wines treated with the PS extracts and CM after one month (T1) and twelve months (T12) of bottling. Table 2 shows the OAV values, odor thresholds, and descriptors [26,27,28,29,30] of the volatile compounds with OAV > 0.2. Twenty-nine volatile compounds were detected and organized into seven different chemical families: higher alcohols, with 1-propanol, isobutanol, 2-methyl-1-butanol, 3-methyl-1-butanol, and 2-phenylethyl alcohol; C6 alcohols, represented by 1-hexanol, *E*-3-hexen-1-ol, *Z*-3-hexen-1-ol, and benzyl alcohol; ethyl esters, with ethyl butyrate, ethyl hexanoate, ethyl octanoate, ethyl decanoate, ethyl-2-methylbutyrate, ethyl isovalerate, and ethyl lactate; acetates, comprising propyl acetate, isobutyl acetate, isoamyl acetate, hexyl acetate, and β-phenethyl acetate; volatile acids, represented by isovaleric acid, hexanoic acid, octanoic acid, and decanoic acid; phenol volatiles, comprising 4-vinylguaicol and 4-vinylphenol; terpenes, formed by linalool and α-terpineol. 

The one-way ANOVA results revealed that the addition of the PS fractions significantly affected the volatile contents of Viura samples both after one month and twelve months of bottling (T1 and T12, respectively).

Higher alcohols were the major chemical family in Viura wines. The use of PS extracts did not produce differences in the content of total higher alcohols after one month of bottling (T1). Only 1-propanol and isobutanol showed significant differences, but both showed OAV values below their odor thresholds (Table 2) and did not contribute to the flavor of the wine. These findings were consistent with those obtained by our research group, who observed that the addition of RG-II-rich extracts and inactivated commercial yeasts rich in MP did not modify the content of higher alcohols in *Albillo* and *Verdejo* wines [16]. However, after twelve months of bottling (T12), the addition of PS extracts reduced the content of total higher alcohols compared to the control wine. In fact, grape-derived PS extracts (WPP and DWRP), rich in RG-II and minor PRAG, reduced the content of higher alcohols more effectively than CM rich in MP. The effect of wine PS on wine volatile compounds has been analyzed by several authors, showing contradictory results on many occasions [6]. Many studies are carried out in model systems and not in real wine samples, and PSs are described to affect aroma volatility, either indirectly “by changing the viscosity of the media” or by direct interaction with volatile compounds, with these interactions being dependent on the physico-chemical structure of the volatile molecules [5] and probably on the physico-chemical composition of the PS involved. Therefore, it seems that the protein part of the PS, such as in MP or PRAG polysaccharides, affects to a greater extent the glycosidic parts regarding the interactions with volatile compounds [13,31], which are mainly hydrophobic interactions and hydrogen binding. Furanic compounds, volatile phenols and aldehydes, and cyclic alcohols (2-phenyl ethanol), all with low hydrophobicity and a flat structure, are retained by the MP since π-π stacking occurs between the aromatic/furanic ring of the compounds and the protein part of the mannoprotein. On the other hand, MP, PRAG, and RG-II can form stable colloids in solution with tannins and protein aggregates [32]. Hence, the reduction in some aromatic compounds could also be attributed to an indirect effect of PS. Higher alcohols would precipitate by the formation of haze and by interactions with wine proteins and phenolics, resulting in hydrophobic interactions, hydrogen bonds, and/or π-π stacking [33,34]; however, the indirect interactions are mainly theorical and need to be contrasted in future studies [35]. The higher decreasing effect of higher alcohols in Viura wines treated with WPP after twelve months could also be attributed to hydrophobic interactions, hydrogen bonds, and/or π-π stacking of the RG-II glycosidic groups, with the largest aromatic family being found in Viura wines. Most higher alcohols are related to herbaceous aromas with pungent and strong notes. Therefore, the use of PS extracts in this study had positive effects on the aroma of the wine.

Wines treated with DWRP and CM increased the contents of C6 alcohols after one month of bottling (T1), while WPP had no effect. After twelve months of bottling (T12), no differences were observed among these aromatic compounds, except for benzyl alcohol, which presented higher concentrations in those wines treated with PS extracts, but it was below its OAV in all the wines (Table 2). Since WPP had no effect on C6 alcohols at T1, and was mainly composed of RG-II (74.7%) [23], the retention effect of DWRP and CM at T1 could be attributed to the protein part of the PRAG in DWRP extract and MP in CM. On the other hand, the loss of the retention effect after twelve months could be due to the fact that these interactions between these aromatic compounds and PS are reversible, which means that the aromatic compounds can be gradually released by the formation of colloidal barriers or absorbing free ethanol molecules to break up the Marangoni effect in wines with an ethanol degree >5% [15,31,35]. Higher MP (or proteic PS) contents could produce higher retention effects [31,36].

C_6_–C_10_ fatty acids are usually related with unpleasant aromas (Table 2); however, they can prevent the hydrolysis of esters [37]. Those wines treated with DWRP and CM extracts presented higher retention of these volatile compounds after one month of aging, which can be due to the higher hydrophobicity of fatty acids because of their carbonyl and hydroxyl groups that can react with some PS as MP [35] and RG-II, which also has been related to the increase in fatty acids in solution [15]. However, no significant differences were observed in the concentrations of the fatty acids between wines treated with PS extracts and control wines after twelve months of bottling. 

Regarding the rest of volatile families, the addition of CM, and specially DWRP extracts, increased the contents of ethyl esters, acetates, volatile phenols, and terpenes after one month of bottling (T1), and presented a great retention effect of most of these volatile compounds after twelve months (T12), since their contents were significantly higher than in the control wines. The use of WPP extracts increased the concentration of total terpenes at T1, related to varietal odorants in wine [38], as well as total ethyl esters and terpenes at T12. The addition of CM increased the content of total ethyl esters, acetates, and terpenes both at T1 and T12. The addition of DWRP extracts increased the content of total ethyl esters, acetates, phenols, and terpenes both at T1 and T12. 

Our results indicated that PS extracts had a satisfactory retention effect on compounds related to fruity aromas [28], as ethyl octanoate and ethyl hexanoate, which presented some of the highest OAVs of the Viura wines (Table 2), and isoamyl acetate and β-phenethyl acetate, providing banana and apple notes to the wine [28]. However, WPP had a lower retention effect than DWRP and CM on most of the volatile compounds.

The higher retention effect of CM and DWRP extracts could be attributed to the proteic part of the MP and PRAG, which would directly interact with the hydrophobic part of the volatile compounds [5] or would form stabilized colloids in solution with phenolic compounds [39,40,41], which retain and protect the aromatic compounds in wine [35]. Moreover, the RG-II molecule have also shown a stabilization effect of these colloids [32], avoiding the release of the aromatic compounds. The retention effect of volatile compounds with planar structures and saturated bonds, as volatile phenols and terpenes, could be due to interactions of the RG-II through hydrophobic interactions or by the proteic part of the PRAG content by H-bonds and π-π stacking [15,35,36] by forming stable colloids in solution.

Considering the results obtained in this study, it can be concluded that grape-derived PS extracts, mainly DWRP, are a good modulator of wine aroma in Viura wines. Generally, those extracts with PS with higher protein contents (DWRP and CM) presented higher retention effects on the volatile compounds. In this sense, more studies are needed to understand the interactions between the molecular compounds of the wines and the effects of the different wine polysaccharides in a different wine matrix. This will allow oenologists to understand the effect of yeast and grape-derived polysaccharides in the winemaking process to improve wine quality.

### 2.3. Phenolic Composition of Viura Wines Treated with PS Extracts and CM

Table 3 shows the effect of the PS extracts and CM on the monomeric phenolic compounds after one month (T1) and twelve months (T12) of bottling. The extracts, WPP, DWRP, and CP, reduced the concentration of total monomeric phenolics in Viura wines at both times. These results agreed with those previously reported by our workgroup [16], who observed that WPP extracts reduced the phenolic content in *Verdejo* wines during deposit storage, and with research which has reported that MPs interact with phenolic compounds, reducing the content of flavonoids in wine [42,43,44,45]. The addition of WPP and DWRP produced a loss of 9% of total monomeric phenolic after one month of bottling. After 12 months of bottling, this reduction was around 19% for WPP, DWRP, and CM. These results do not demonstrate a gradual desorption of the monomeric polyphenols adsorbed by the PS extracts and CM added to the wine, as described by [35], who points out that non-covalent and reversible MP-polyphenol interactions can present a gradual desorption of polyphenols to wine during aging. Probably, most of the monomeric polyphenols joined irreversibly with the PS of WPP, DWRP extracts, and CM. This loss is probably favored by the molecular weight of the PS of the grape-derived extracts and CMs that are susceptible to interact with the monomeric molecules of the polyphenols. WPP and DWRP extracts were mainly composed of medium- and low-molecular-weight PS, and CM was composed of both low- and high-molecular-weight PS [24]. However, it is important to note that the content of monomeric phenolics in white wines is low. Therefore, studies in red wines with higher contents of these compounds are needed to confirm these results. A Multivariate analysis of variance (MANOVA) of phenolic compounds of Viura wines after one month (T1) and twelve months of bottling (T12) with the Percentage of variance attributable (%) of the independent effect of Aging time and PS extract, and the interaction of both (Aging × PS extract) can be consulted in the supplementary material (Table S1).

### 2.4. Proanthocyanins of Viura Wines Treated with PS Extracts and CM

Table 4 shows the total proanthocyanidin (PA) content and mean degree of polymerization (mDP), as well as the percentages of epicatechin, catechin, and epicatechin gallate terminal subunits in Viura wines treated with the PS extracts and CM at T1 and T12. The PA content was similar to that described by [46] in Viura wines. No statistically significant differences were obtained for total PA concentration at T1. The % of catechin, the primary terminal subunit in the grape skin [47], and epicatechin was higher in the wines treated with the PS extracts, except for % (+) catechin in the WPP wine. The mDP, which is an indicator of the wine sensory properties of bitterness and astringency [48], significantly decreased in the wines treated with the grape-derived PS extracts (WPP and DWRP) and CM. 

After 12 months of bottle aging (T12), the use of both grape-derived PS extracts and CM produced a loss of PA (Table 4). The addition of WPP and DWRP produced a similar reduction in the PA content compared to CM (~22–29%). In the bibliography, a reduction in the wine astringency produced by MP is described due to a precipitation effect. Therefore, the flocculant effect can lead to coaggregation and precipitation of MP–flavanol complexes in wines, and a decrease in flavanols [49,50,51]. In the same way, the high content of RG-II dimmer in the WPP and DWRP extracts and its interaction with flavanols to form complexes explains the decrease in PA in the wines. Riou et al. [52] reported that RG-II strongly enhanced the mean apparent diameter of these complexes with time, suggesting co-aggregation between RG-II dimer and tannin. After 12 months of bottling, wines treated with WPP and DWRP had lower contents of PA than control wines, probably due to the size increase and precipitation of the PA-RG-II complexes, corroborating the results obtained by [52]. As observed in T1, the use of grape-derived PS extracts reduced the mDP of PA, obtaining similar effects between DWRP and CM. It can be concluded that the grape-derived PS extracts can potentially reduce the astringency perception since they reduced both the PA content and the mDP of the PA, related to the astringency and bitter sensations [48]. Therefore, these extracts have a good potential to be considered as finning agents to modulate wine astringency and bitterness, which is especially important in white wines with an undesirable excess of bitterness or astringency sensations.

### 2.5. Sensory Characteristics of Viura Wines Treated with PS Extracts and CM

**Figure 1:**
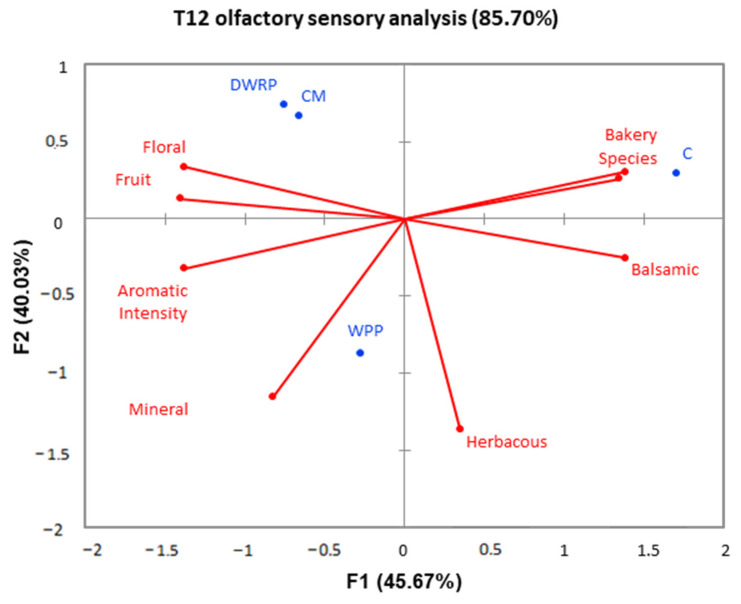
Generalized Procrustes analysis (GPA) of the mean ratings for olfactory phase in Viura wines after twelve months of bottling. C: control wines; WPP: Wine-Purified Polysaccharides; DWRP: Distilled Washing Residues Polysaccharides; CM: Commercial Mannoproteins.

**Figure 2:**
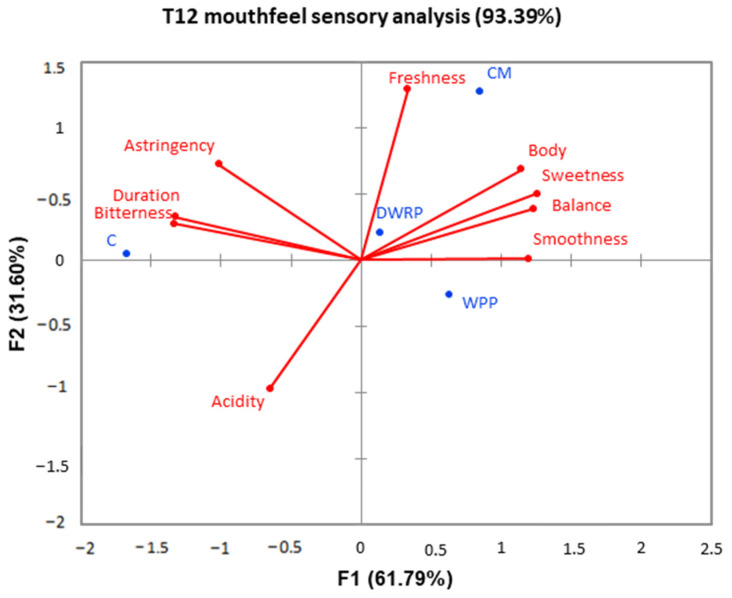
Generalized Procrustes analysis (GPA) of the mean ratings for gustative phase in Viura wines after twelve months of bottling. C: Control wines; WPP: Wine-Purified Polysaccharides; DWRP: Distilled Washing Residues Polysaccharides; CM: Commercial Mannoproteins.

The Viura wines were analyzed in terms of their gustative and olfactory attributes after twelve months of aging (T12), and a generalized Procrustes analysis (GPA) consensus configuration is presented in Figure 1 and Figure 2, respectively. 

The primary two factors of the olfactory GPA space accounted for 85.7% of the overall variation (Figure 1), and the CM, WPP, and DWRP wines were well differentiated from the control wines. The wines were differentiated according to their aromatic intensity, floral, fruit, mineral, balsamic, bakery, species, and herbaceous notes. CM and DWRP wines were very close in the GPA space, indicating that both produced the same effect on the wine’s olfactory characteristics. The use of both WPP and DWRP extracts at wine finning increased the aromatic intensity of the wines and their floral and fruit notes, which is in good agreement with the data obtained for the volatile compounds. However, the wines treated with the WPP extract showed a different behavior. The use of WPP extracts also increased the aromatic intensity and floral and fruit notes in comparison with the control wines, but these wines were also characterized by herbaceous notes.

The average space of gustatory attributes is shown in Figure 2, which explains 93.4% of the entire variation. The wines with the addition of grape-derived PS extracts and CM were again separated from the control wine in the GPA space. Therefore, wines were differentiated according to their astringency, bitterness, duration, freshness, body, sweetness, balance, smoothness, and acidity. The addition of commercial mannoproteins increased freshness sensations as well as body, sweetness, balance, and smoothness, and reduced acidity perception. These results agreed with the bibliography data [40] and with the use recommended by the commercial manufacturers for these purified products. This paper describes, for the first time, the potential use of grape-derived polysaccharides on the gustatory and olfactory sensations of white wines. Our results demonstrated that grape-derived PS extracts were as good modulators of mouthfeel sensations as CM. Both WPP and DWRP improved body, sweetness, balance, and smoothness, and reduced astringent and bitterness sensations. Therefore, tribology, defined as the quantification of friction and lubrication of food–saliva mixtures in the oral mucosa, which is related with astringency, “dryness feeling”, and smoothness in wine perceived by the consumer [53], would be an interesting address in future studies to complement the knowledge of the effect of PS extracts on these parameters that are so important in the quality of wines.

In general, both grape-derived PS extracts improved the olfactory and mouthfeel characteristics of the wines, proving to be a good alternative to commercial mannoproteins as modulators of white wine flavor compounds. Moreover, the extract obtained from the distilled washing residues of wine pomaces (DWRPs) showed to have even more positive effects than the purified PS extract obtained from wine (WPP). Also considering that the process for the extraction of the DWRP is much simpler and less time-consuming, our results explore the possibility of obtaining valuable polysaccharides from distilled washing residues, which could promote its valorization as a by-product, contributing to the circular economy. A correlation between oenological and phenolic parameters and sensory results can be consulted in the supplementary material (Table S2).

## 3. Material and Methods

### 3.1. Polysaccharide Extracts and Commercial Mannoproteins

As previously described [24], a first fraction of polysaccharides was obtained from the freeze-dried polysaccharides extracted from a *Carignan noir* wine by two successive steps of anion-exchange [54]. Polysaccharides were loaded on a Fractogel EMD DEAE 650 (M) (Merck, Darmstadt, Germany) column (18 × 24 cm^2^). An unbound fraction was recovered, and the bound polysaccharides were eluted as described [24]. The fraction eluted by 50 mM of NaCl on the Fractogel EMD DEAE 650 was loaded on a concanavalin A-Sepharose (Pharmacia, Sweden) column-equilibrated 50 mM sodium acetate buffer pH 5.6 containing 150 mM NaCl, 1 mM CaCl_2_, 1 mM MgCl_2_, and 1 mM MnCl_2_, and the unbound fraction was collected, dialyzed against water, freeze dried, and named WPP (Wine-Purified Polysaccharides). A second fraction of polysaccharides was obtained from the wash water used by the distillery after draining the distilled wine pomace. It was concentrated, precipitated, and lyophilized as described [24] to obtain the DWRP fraction (Distilled Washing Residues Polysaccharides). A commercial product rich in mannoproteins (CM, commercial mannoproteins) and recommended for wine finning at bottling was supplied by Lallemand Bio S.L. (Logroño, Spain).

These three extracts were characterized in terms of monosaccharide and PS composition, PS molecular weight distribution, and PS purity as described [24]. WPP showed the highest polysaccharide purity (89.7%), followed by CM (66.2%) and DWRP (40.6%) [24]. WPP was mainly composed of RG-II (74.7%) and small amounts of PRAG (14.7%), HG (6.9%), and glycosyl polysaccharides (GP) like celluloses and hemicelluloses (3.2%), and DWRP was mainly composed of RG-II (51.1%), PRAG (26.0%), HG (19.1%), and small amounts of GP (2.7%). CM was mainly composed of MP (74.7%) and glucans (25.3%) [24]. HPSEC-RID was used to determine the molecular weight (Mw) distributions of the PS extracts. WPP was mainly composed of medium-Mw PS (71.2%) and low-Mw PS (28.8%). DWRP presented similar Mw distributions than WPP (53.8% of medium-Mw PS and 46.2% of low-Mw PS). Finally, CM was composed of low- (52.8%) and high-Mw PS (45.6%). 

### 3.2. Winemaking and Trials

A white wine was made from Viura Vitis vinifera L. variety by traditional winemaking in 2018 in a winery of Rioja Qualified Denomination of Origin (D.O.Ca Rioja). Harvest was carried out at optimum maturity (22.9 °Brix, pH 3.35, 6.53 g L^−1^ total acidity as g L^−1^ tartaric acid), and grapes were destemmed-crushed and pressed (BucherVaslin XPro 8, Chalonnes-sur-loire, France). Must was fermented in a stainless-steel deposit at 14 to 16 °C after inoculation with 0.15 g L^−1^ of *Saccharomyces cerevisiae* yeast (Martin Vialate, Magenta, France). Fermentation took 12 days, and finally the wines were cold-settled. The PS extracts were added to the wine 24 h before filtration and bottling. The wines were bottled in green, standard 750 mL wine bottles (Bordelaise) sealed with natural cork stoppers. Bottles were stored in a room under controlled temperature (12–15 °C) and humidity (60–75%) conditions.

Four experiments were carried in triplicate: control wine (without no product addition, C); wine with the addition of purified PS obtained from wine (WPP); wine with the addition of purified PS obtained from the pomace distilled washing residues (DWRP); and wine treated with commercial mannoproteins (CMs). The doses used for the PS extracts and CM were 0.10 g L^−1^. 

### 3.3. Standard Oenological Parameters 

General oenological parameters in the wines were measured according to the official methods described by the International Organization of Vine and Wine [55]: pH, total acidity (g L^−1^ tartaric acid), volatile acidity (g L^−1^ acetic acid), alcohol content (% vol: mL ethanol for 100 mL wine at 20 °C), absorbance at 420 nm, free SO_2_ (mg L^−1^ free sulfur dioxide), and total SO_2_ (mg L^−1^ total sulfur dioxide). Malic acid was analyzed by the autoanalyzer BioSystems Y15 (Biosystem, Barcelona, Spain). 

### 3.4. Analysis of Volatile Compounds

Higher alcohols were quantified by the direct injection of wine in split mode (25:1), using an Agilent 7890A gas chromatograph (Santa Clara, CA, USA) with a flame ionization detector and the chromatographic conditions previously described [56]. The identification was carried out using spectra obtained with commercial standard compounds and from the NIST library. Quantification was carried out following the internal standard quantification method, using the selected quantification ions and chosen IS for each compound as well as the calibration curves described in [56].

Volatile compounds found in lower concentrations in the wine were quantified by headspace solid-phase micro-extraction (autosampler PAL RSI 120) and gas chromatography with mass spectrometer (Agilent 78902B CG coupled to a 5977B MSD). In total, 10 mL of wine was diluted (1:3 with an hydroalcoholic solution and the addition of four internal standards (IS): methyl 2-methylbutyrate, methyl octanoate, heptanoic acid, and 3,4-dimethylphenol) and placed into a 20 mL glass vial with 3.5 g L^−1^ of sodium chloride. The samples were incubated 5 min at 40 °C, and after that the volatiles in the headspace of the vial were extracted with a 1 cm 50/30-μm DVB/Carboxen/PDMS SPME fiber (Supelco) at the same temperature and with an agitation speed of 500 rpm during 60 min. After extraction, the fiber was desorbed for 3 min in the injector at 250 °C using the splitless mode. Chromatographic analyses were carried out with a DB-WAX Ultra Inert capillary column (60 m length, 0.25 mm i.d., and 0.50 mm film thickness, Agilent). The volatile composition of the wines was analyzed in triplicate after one month of bottling (T1) and after twelve months of bottle aging (T12). The odor activity value (OAV) was used to evaluate the potential contribution of a chemical compound to wine aroma. This parameter provides a rough pattern of the sensory importance of odorants by converting quantitative data into sensory information [57]. We have considered that odorants with higher OAVs (>0.2) contribute more strongly to overall aroma. 

### 3.5. Quantification of Monomeric and Polymeric Phenolic Compounds by HPLC-DAD

Monomeric phenolic compounds were determined by using the Agilent 1100 liquid chromatograph equipped with a LiChrospher 100 RP-18 reversed-phase column (0.4 × 25 cm, 5 µm) as described [58]. DAD chromatographs were obtained at 360 nm (flavonols), 320 nm (hydroxybenzoic and hydroxycinnamic acids), and 280 nm (flavanols) with the calibration curves of their respective standards (r^2^ > 0.999) or according to the calibration of the most similar compound standard. Each wine was analyzed in triplicate after one month (T1) and twelve months (T12) of bottling.

The flavan-3-ols and condensed tannins of Viura wines were analyzed following a methodology previously described [59]. A multi-step analytical method with an initial fractionation of wine phenolics by gel permeation chromatography (GPC) with a TSK Toyorpeal gel HW-50F (Tosohaas, Montgomery-ville, PA, USA) packed in a Millipore (Bedford, MA, USA) Vantage L column (120 mm × 12 mm i.d.). Two milliliters (2 mL) of wine was directly applied to the column and the flow rate was regulated at 1 mL min^−1^. The first fraction was eluted with 60 mL of a solution of ethanol/water/trifluoroacetic acid (55:45:0.05, *v*/*v*/*v*), and the second fraction was recovered by elution with 50 mL of acetone/water (60:40, *v*/*v*); the second fractions were taken under dryness under vacuum conditions. 

The dried fractions of wine flavan-3-ols and condensed tannins were solved in 1.5 mL of MeOH and 0.5 mL of phloroglucinol solution made by 0.1 N HCl in MeOH, 50 g L^−1^ phloroglucinol, and 10 g L^−1^ ascorbic acid, were added, and the solution was at 50 °C for 20 min [59]. After the reaction time, 0.5 mL of sodium acetate (40 mM) was added to stop the reaction of the proanthocyanins and was analyzed by HPLC-DAD. Each fined wine was analyzed in triplicates after one month (T1) and twelve months (T12) of bottling.

The flavan-3-ols and condensed tannins of Viura wine were quantitated by high-performance liquid chromatography–diode array detection (HPLC-DAD) in an Agilent modular 1100 liquid chromatograph (Waldbronn, Germany) using an ACE HPLC (5 C18-HL) column (5 µm packing, 200 mm × 46 mm i.d.). Phenolic compounds were eluted at a 1 mL min^−1^ flow rate with solvent A: formic acid/water (2:98, *v*/*v*); solvent B: acetonitrile/water/formic acid (80:18:2, *v*/*v*/*v*). The chromatograms were acquired at 280 nm for the identification and quantitation of flavan-3-ols and condensed tannins. The total proanthocyanidin content (PA) was calculated as the sum of extension subunits (phloroglucinol adducts) and terminal subunits (catechin, epicatechin, and epicatechin gallate). The apparent mean degree of polymerization (mDP) was calculated as the sum of all subunits divided by the sum of the terminal subunits.

### 3.6. Sensory Analysis

The sensory analysis was carried out by 14 expert tasters (6 males and 8 females, 25–52 years old). Firstly, the tasters selected the descriptors to be used in the sensory analysis. In a second session, wine samples were scored using a structured numerical scale of six points (0 represented no intensity and 5 the highest intensity) according to UNE-87-020-93 Standard [60]. The samples were presented in random order in standard glasses and codified with a three-digit number. The data of sensory analysis after twelve months of bottling (T12) are presented.

### 3.7. Statistical Analyses

A one-way analysis of variance (ANOVA) with the Duncan post hoc testing was carried out using SPSS Statics 23 (IBM Corp., Armonk, NY, USA). Generalized Procrustes analysis (GPA) used for the sensory data was made with the XLSTAT 2022.1 Software (Addinsoft Inc., New York, NY, USA). 

## 4. Conclusions

This paper describes the use of purified grape-derived polysaccharides from red wine (WPP) and winemaking by-products (DWRP: Distilled Washing Residues Polysaccharides) as fining agents at the bottling of white Viura wines.

Both extracts, rich in rhamnogalacturonans-II, showed themselves to be good modulators of wine aroma. Except for higher alcohols and volatile acids, both extracts increased the content of all volatile families after one and twelve months of bottle aging. The results also showed a positive contribution in wine astringency and bitterness, related to a reduction in the proanthocyanidin content and its mean degree of polymerization. Moreover, both extracts improved the olfactory and mouthfeel characteristics of the wines, proving to be a good alternative to commercial mannoproteins as modulators of white wine flavor compounds. DWRP extract showed more positive effects on the overall wine aroma and sensory properties, opening the possibility of obtaining valuable polysaccharides from distilled washing residues of wine pomaces. More studies are needed to understand the interactions between the molecular compounds of the wines and the effects of the different wine polysaccharides extracts. 

## Figures and Tables

**Table 1:** Concentration of volatile compounds (µg L^−1^) ^a^ of Viura wines treated with the PS extracts and CM after one (T1) and twelve months of bottling (T12).

	T1					T12				
Compounds	C ^b^	WPP ^b^	DWRP ^b^	CM ^b^	F-Value	C ^b^	WPP ^b^	DWRP ^b^	CM ^b^	F-Value
1-propanol	33,643 (1522.0) b	37,529 (1442.0) c	32,195 (1180.8) a,b	30,672 (200.4) a	17.807 ***	32,796 (1171.0) c	27,407 (876.1) a	29,552 (1246.6) b	29,756 (154.1) b	31.726 ***
Isobutanol	1259.3 (39.6) b	1040.9 (71.8) a	1334.4 (62.6) b	1324.7 (58.7) b	15.938 ***	22,424 (1125.7) c	18,340 (1603.0) a	20,079 (1068.4) b	20,687 (543.8) b	12.927 **
2-Methyl-1-butanol	29,536 (2762.0)	29,560 (1583.1)	29,834 (1103.0)	27,687 (831.9)	0.972 (ns)	27,447 (2124.9) b	20,988 (4947.0) a	24,600 (2307.3) a,b	26,457 (639.9) b	5.598 *
3-Methyl-1-butanol	28,933 (2015.0)	25,780 (1510.9)	26,576 (2295.3)	28,253 (2114.2)	3.073 (ns)	158,137 (8747.6) b	125,476 (20,626) a	143,312 (8373.6) b	149,973 (3206) b	7.953 **
2-Phenylethanol	17,197 (104.3)	15,681 (866.3)	18,104 (1736.7)	15,847 (323.5)	3.987 (ns)	23,143 (1357.6) a	24,122 (963.1) b	22,406 (243.8) a	24,360 (126.1) b	20.059 ***
TOTAL HIGHER ALCOHOLS	110,569 (6442.9)	109,592 (4431.8)	108,044 (5858.4)	103,784 (3042.8)	3.380 (ns)	263,948 (14,526) c	216,335 (25,337) a	239,951 (13,239) b	251,234 (4109.5) bc	9.723 **
1-Hexanol	444.9 (20.0) a	481.3 (22.9) a	986.7 (100.6) b	1037.8 (134.3) b	41.750 ***	960.9 (22.2)	952.4 (13.4)	953.2 (15.7)	960.9 (11.9)	0.506 (ns)
€-3-Hexenol	57.8 (4.2) a	47.0 (2.8) a	132.8 (8.7) c	78.0 (6.8) b	118.549 ***	95.6 (6.9)	94.1 (2.1)	98.8 (0.4)	97.7 (8.7)	0.831 (ns)
(Z)-3-Hexenol	181.2 (8.5) a	149.2 (6.0) a	416.2 (53.7) c	244.3 (30.7) b	43.235 ***	485.8 (7.8)	488.3 (1.3)	491.4 (3.0)	492.8 (6.0)	2.080 (ns)
Benzyl alcohol	198.5 (8.4) b	198.5 (7.3) b	213.3 (15.7) b	107.4 (10.2) a	59.554 ***	153.2 (4.3) a	167.8 (8.4) b	177.3 (2.4) c	173.3 (3.8) bc	25.325 ***
TOTAL C6 ALCOHOLS	882.4 (23.7) a	876.0 (35.0) a	1749.0 (178.7) c	1467.5 (182.0) b	33.611 ***	1695.5 (18.8)	1703.2 (18.6)	1720.6 (21.6)	1724.6 (5.3)	3.691 (ns)
Ethyl butyrate	54.0 (2.4) a	54.0 (7.9) a	110.3 (5.7) b	62.6 (10.7) a	40.579 ***	213.9 (6.7) a	254.5 (0.2) c	232.8 (1.4) b	263.4 (4.6) d	175.091 ***
Ethyl hexanoate	722.6 (88.1) a	740.5 (31.8) a	1474.6 (120.9) b	861.0 (72.4) a	52.911 ***	353.4 (10.2) a	374.9 (7.2) b	354.8 (0.6) a	403.4 (3.1) c	79.376 ***
Ethyl octanoate	1058.2 (89.8) a	1003.7 (32.3) a	2069.5 (422.9) b	1373.3 (117.8) a	14.281 ***	266.5 (4.1) b	256.3 (1.9) a	252.0 (3.3) a	293.9 (3.8) c	188.401 ***
Ethyl decanoate	237.8 (40.5) a	210.4 (13.2) a	579.1 (122.7) c	388.0 (32.9) b	19.152 ***	32.2 (2.9) a	33.5 (0.5) a	32.0 (1.6) a	38.4 (1.8) b	14.596 ***
Ethyl-2-methylbutyrate	10.6 (0.2) a	10.1 (0.3) a	12.2 (0.4) b	10.1 (0.1) a	38.058 ***	7.2 (0.2) a	7.0 (0.1) a	8.0 (0.6) b	7.3 (0.2) a	10.679 **
Ethyl isovalerate	22.0 (2.1)	23.6 (2.1)	24.5 (1.3)	21.1 (1.5)	2.213 (ns)	17.8 (2.3)	17.6 (3.5)	19.3 (0.2)	16.5 (2.0)	1.274 (ns)
Ethyl lactate	1067.1 (40.2) a	992.9 (30.5) a	2522 (379.8) c	1577.9 (247.2) b	28.642 ***	21,806.8 (211.9) a	22,412.9 (217.2) b	22,626.0 (126.8) b,c	22,861.0 (96.4) c	21.994 ***
TOTAL ETHYL ESTERS	3172.3 (263.3) a	3035.2 (100.8) a	6792.1 (594.0) c	4294.0 (285.3) b	25.530 ***	22,697.7 (212.3) a	23,356.6 (217.2) b	23,524.9 (126.8) b	23,883.9 (96.1) c	25.116 ***
Propyl acetate	565.2 (2.0) a	571.5 (31.1) a	1131.6 (56.6) c	712.0 (84.1) b	75.742 ***	34.9 (1.2)	34.4 (0.5)	32.8 (0.1)	35.1 (1.4)	3.771 (ns)
Isobutyl acetate	367.0 (72.5) a,b	334.8 (33.3) a	754.9 (15.4) c	412.4 (28.7) b	155.111 ***	27.4 (2.0) a	28.6 (2.2) a	25.8 (0.6) a	35.8 (4.9) b	7.058 *
Isoamyl acetate	2554.5 (250.1) a,b	2272.8 (66.0) a	5156.1 (173.8) c	3110.6 (522.5) b	55.001 ***	965.1 (22.3) a	963.8 (5.6) a	1011.6 (28.3) b	1028.3 (40.3) b	4.368 *
Hexyl acetate	384.2 (53.0) a	310.2 (16.7) a	726.2 (86.8) b	374.1 (61.1) a	29.512 ***	37.3 (1.9) a	40.5 (0.3) c	38.1 (0.1) a	38.8 (0.4) b,c	5.596 *
β-Phenylethyl acetate	543.3 (1.4) a,b	434.2 (48.2) a	1081.7 (50.6) c	625.9 (95.5) b	69.424 ***	69.8 (0.6) a	78.0 (0.2) c	78.4 (1.4) c	77.5 (1.2) c	52.655 ***
TOTAL ACETATES	4414.2 (279.0) a	3923.5 (98.8) a	8850.4 (355.3) c	5235 (716.2) b	69.250 ***	1134.5 (17.1) a	1145.2 (6.9) a	1186.6 (28.9) b	1215.5 (48.1) b	4.838 *
Isovaleric acid	38.8 (0.1) a	33.5 (2.4) a	53.8 (8.7) b	58.9 (9.6) b	10.024 **	365.3 (5.0)	362.5 (0.8)	365.1 (7.2)	376.1 (12.8)	0.22 (ns)
Hexanoic acid	1221.7 (17.5) a	1071.0 (109.8) a	1630.8 (176.2) b	1610.2 (217.9) b	10.403 **	4384.4 (17.1) b	4342.0 (3.4)	4318.0 (44.0)	4302.1 (47.1)	0.072 (ns)
Octanoic acid	2574.9 (12.8) b	1989.3 (267.2) a	3062.8 (190.9) c	3076.9 (306.5) c	15.690 ***	4.035.4 (10.0) a	4140.3 (59.4)	4040.2 (6.2)	4131.6 (89.6)	0.078 (ns)
Decanoic acid	41.2 (10.2) a,b	37.2 (3.1) a	66.2 (6.4) c	60.5 (19.4) b,c	4.576 *	301.7 (12.2) b	297.3 (5.8)	296.6 (1.9)	304.8 (7.5)	0.555 (ns)
TOTAL ACIDS	3876.6 (40.6) b	3131.0 (382.5) a	4813.6 (382.2) c	4806.5 (553.4) c	13.222 **	9086.7 (34.3)	9141.9 (69.4)	9020.2 (59.3)	9114.5 (141.9)	0.403 (ns)
4-vinylguaiacol	332.5 (46.3) a	299.0 (7.2) a	662.8 (116.9) b	345.7 (21.3) a	21.176 ***	121.4 (1.3) a	128.3 (6.8) a	141.3 (8.3) b	126.7 (4.3) a	23.578 ***
4-vinylfenol	267.5 (45.6) a	262.7 (10.3) a	664.1 (47.9) c	373.4 (22.6) b	85.457 ***	46.9 (0.6) a	47.0 (0.6) a	56.7 (0.2) b	49.2 (0.3) a	7.189 ***
TOTAL PHENOLS	600.0 (91.9) a	561.7 (8.8) a	1326.9 (158.9) b	719.1 (43.9) a	40.515 ***	168.3 (1.4) a	175.3 (6.8) a	198.0 (8.3) b	175.9 (4.3) a	17.713 ***
Linalool	37.1 (1.9) a	45.9 (1.9) b	42.8 (2.2) b	44.5 (1.8) b	11.642 ***	27.5 (2.3) a	35.1 (2.5) b	34.4 (0.7) b	34.3 (1.8) b	19.486 ***
α-Terpineol	6.2 (0.2) a	6.5 (0.2) a	6.9 (0.1) b	6.3 (0.1) a	11.500 **	2.8 (0.1) c	2.6 (0.1) b	3.8 (0.2) d	2.3 (0.1) a	163.189 ***
TOTAL TERPENES	43.3 (1.9) a	52.4 (1.9) b	49.7 (2.3) b	50.8 (1.8) b	12.057 ***	30.3 (2.3) a	37.6 (2.6) b	38.2 (0.8) b	36.6 (1.8) b	19.233 ***

^a^ Mean values are shown (n = 3). Different letters (a, b, c, and d) in the same row indicate significant differences (*p* < 0.05). Level of significance: *, **, and *** indicate significance at *p* < 0.05, at *p* < 0.01, and at *p* < 0.001, respectively. ^b^ Control wine (C) and wines treated with the PS extracts. WPP: Wine-Purified Polysaccharides, DWRP: Distilled Washing Residues Polysaccharides; CM: Commercial Mannoproteins. ns: not significant.

**Table 2:** Odor activity values (OAVs >0.2) ^a^ of Viura wines treated with the PS extracts and CM after one (T1) and twelve months of bottling (T12).

				T1					T12				
Compounds	Odor Descriptor	Odor Threshold (µg L^−1^)	Reference	C ^b^	WPP ^b^	DWRP ^b^	CM ^b^	F-Value	C ^b^	WPP ^b^	DWRP ^b^	CM ^b^	F-Value
Isobutanol	Alcohol, solvent, green, bitter	75,000	[25]						0.3	0.2	0.3	0.3	3.167 (ns)
2-Methyl-1-butanol	Alcohol	30,000	[25]	1.0	1.0	1.0	0.9	0.972 (ns)	0.9 b	0.7 a	0.8 b	0.9 b	7.667 *
3-Methyl-1-butanol	Alcohol	7000	[26]	4.1	3.7	3.8	4.0	3.073 (ns)	5.3 c	4.2 a	4.8 b	5.0 b	7.921 **
2-Phenylethanol	Roses, honey	14,000	[27]	1.2	1.1	1.3	1.1	3.987 (ns)	1.6 a	1.7 b	1.6 a	1.7 b	17.000 ***
(E)-3-Hexenol	Green, floral	400	[27]	0.1 a	0.1 a	0.3 c	0.2 b	118.549 ***	0.2	0.2	0.2	0.2	0.667 (ns)
(Z)-3-Hexenol	Green, floral	400	[27]	0.5 a	0.4 a	1.0 c	0.6 b	43.235 ***	1.2	1.2	1.2	1.2	1.383 (ns)
Ethyl butyrate	Papaya, apple	20	[26]	2.7 a	2.7 a	5.5 b	3.1 a	40.579 ***	10.7 a	12.7 c	11.6 b	13.2 d	219.933 ***
Ethyl hexanoate	Apple, fruity, sweetish	14	[26]	51.6 a	52.9 a	105.3 b	61.5 a	52.911 ***	25.2 a	26.8 b	25.3 a	28.8 c	74.772 ***
Ethyl octanoate	Apple, fruity, sweetish	5	[26]	211.6 a	200.7 a	413.9 b	274.7 a	14.281 ***	53.3 b	51.3 a	50.4 a	58.8 c	173.479 ***
Ethyl decanoate	Grape	200	[27]	1.2 a	1.1 a	2.9 c	1.9 b	19.152 ***					
Ethyl 2-methylbutyrate	Fruity, strawberry, apple, blackberry	2	[30]	5.3 a	5.0 a	6.1 b	5.1 a	38.058 ***	3.6 a	3.5 a	4.0 b	3.7 a	10.563 **
Ethyl isovalerate	Fruity	3	[28]	7.3	7.9	8.2	7.0	2.213 (ns)	5.9	5.9	6.4	5.5	1.275 (ns)
Isoamyl acetate	Banana, apple	30	[28]	85.2 a,b	75.8 a	171.9 c	103.7 b	55.001 ***	32.2 a	32.1 a	33.7 a	34.3 b	4.873 *
β-Phenylethyl acetate	Banana	250	[27]	2.2 a,b	1.7 a	4.3 c	2.5 b	69.424 ***	0.3	0.3	0.3	0.3	1.036 (ns)
Isovaleric acid	Cheese	33	[27]	1.2 a	1.0 a	1.6 b	1.8 b	10.024 **	10.9	10.9	10.9	11.3	0.221 (ns)
Hexanoic acid	Cheese, fatty	3000	[29]	0.4 a	0.4 a	0.5 b	0.5 b	10.403 **	1.5	1.4	1.4	1.4	0.072 (ns)
Octanoic acid	Cheese, fatty, rancid	1000	[29]	2.6 b	2.0 a	3.1 c	3.1 c	15.690 ***	4.0	4.1	4.0	4.1	0.078 (ns)
4-vinylguaiacol	Clove, curry	40	[28]	8.3 a	7.5 a	16.6 b	8.6 a	21.176 ***	3.0 a	3.2 a	3.5 b	3.2 a	23.578 ***
4-vinylphenol	Smoky, almond	180	[28]	1.5 a	1.5 a	3.7 c	2.1 b	85.457 ***	0.3	0.3	0.3	0.3	0.423 (ns)
Linalool	Floral, citrus	25	[25]	1.5	1.8	1.7	1.8	1.833 (ns)	1.1	1.4	1.4	1.4	1.129 (ns)

^a^ Mean values are shown (n = 3). Different letters (a, b, c, and d) in the same row indicate significant differences (*p* < 0.05). Level of significance: *, **, and *** indicate significance at *p* < 0.05, at *p* < 0.01, and at *p* < 0.001, respectively. ^b^ Control wine (C) and wines treated with the PS extracts. WPP: Wine-Purified Polysaccharides, DWRP: Distilled Washing Residues Polysaccharides; CM: Commercial Mannoproteins. ns: not significant.

**Table 3:** Concentration of monomeric phenolic (mg L^−1^) ^a^ of Viura wines treated with the PS extracts and CM after one (T1) and twelve months of bottling (T12).

	T1					T12				
Flavonoids ^c^	C ^b^	WPP ^b^	DWRP ^b^	CM ^b^	F-Value	C ^b^	WPP ^b^	DWRP ^b^	CM ^b^	F-Value
Flavonols										
Myricetin-3-gal	1.30 (0.02)	nd	nd	nd	-	0.91 (0.01)	nd	nd	nd	-
Myricetin-3-glcU	1.92 (0.04)	1.79 (0.19)	1.50 (0.32)	1.40 (0.42)	2.559 (ns)	1.34 (0.03)	1.25 (0.12)	1.05 (0.2)	0.98 (0.27)	3.095 (ns)
Myricetin-3-glc	0.95 (0.01) b	0.30 (0.02) a	1.39 (0.01) d	0.99 (0.10) c	356.043 ***	0.67 (0.01) b	0.21 (0.01) a	0.97 (0.01) c	0.69 (0.06) b	433.25 ***
Quercetin-3-glcU	1.80 (0.04) b	1.10 (0.32) a	1.88 (0.21) b	4.04 (0.52) c	31.623 ***	1.26 (0.03) b	0.77 (0.2) a	1.32 (0.13) b	2.83 (0.33) c	39.401 ***
Quercetin-3-glc	1.73 (0.04) a	3.33 (0.74) b	1.24 (0.34) a	1.06 (0.19) a	18.696 ***	1.21 (0.03) a	2.33 (0.47) b	0.87 (0.22) a	0.74 (0.12) a	22.619 ***
Free kaempferol	1.86 (0.04) a	5.48 (1.06) c	2.86 (0.34) a,b	4.60 (0.78) b	14.731 ***	1.30 (0.03) a	3.84 (0.68) c	2.00 (0.22) a	3.22 (0.50) b	17.796 ***
Free syringetin	3.28 (0.91)	nd	nd	nd	-	2.30 (0.58)	nd	nd	nd	-
Isorhamnetin	1.63 (0.02) d	0.28 (0.03) a	0.36 (0.04) b	0.41 (0.04) c	1443.579 ***	1.14 (0.01) d	0.20 (0.02) a	0.25 (0.03) b	0.29 (0.03) c	1483.778 ***
Total myricetin	4.17 (0.05) c	2.09 (0.19) a	2.89 (0.32) b	2.39 (0.43) b	46.759 ***	2.92 (0.03) c	1.46 (0.12) a	2.02 (0.2) b	1.67 (0.28) b	56.42 ***
Total quercetin	3.53 (0.06) a,b	4.43 (0.81) b	3.12 (0.40) a	5.10 (0.55) b	4.190 *	2.47 (0.04) a,b	3.10 (0.51) b,c	2.19 (0.26) a	3.57 (0.35) c	5.029 *
Total kaempferol	1.86 (0.04) a	5.48 (1.06) c	2.86 (0.34) a,b	4.60 (0.78) b	14.731 ***	1.30 (0.03) a	3.84 (0.68) c	2.00 (0.22) a	3.22 (0.50) b	17.796 ***
Total syringetin	3.28 (0.91)	nd	nd	nd	-	2.30 (0.58)	nd	nd	nd	-
Total Flavonols	14.47 (0.91) c	12.28 (1.35) b	9.23 (0.62) a	12.50 (1.05) b	24.269 ***	10.13 (0.58) c	8.60 (0.86) b	6.46 (0.39) a	8.75 (0.67) b	29.347 ***
Flavanols										
Epigallocatechin	7.08 (0.84) b	3.85 (0.78) a	3.54 (0.65) a	4.44 (0.71) a	17.396 ***	3.03 (0.13)	2.99 (0.09)	2.99 (0.13)	2.94 (0.08)	0.078 (ns)
Catechin	17.96 (1.32)	17.79 (1.47)	17.92 (1.67)	18.98 (1.79)	0.021 (ns)	29.83 (2.34)	29.86 (2.41)	30.22 (2.22)	29.52 (2.31)	0.019 (ns)
Epicatechin	14.38 (1.44)	16.20 (1.09)	16.54 (1.43)	17.40 (2.01)	1.440 (ns)	16.58 (1.54) c	2.48 (0.52) a	4.24 (0.37) b	4.14 (0.42) b	172.286 ***
Total Flavanols	39.42 (2.13)	37.84 (1.99)	38.00 (2.29)	40.82 (2.79)	0.871 (ns)	49.44 (2.80) b	35.33 (2.47) a	37.45 (2.25) a	36.60 (2.35) a	11.553 **
Hydroxybenzoic Acids (HBAs)										
Gallic acid	8.70 (1.02)	7.70 (0.85)	7.55 (0.67)	7.98 (0.77)	1.253 (ns)	5.02 (0.76) a	7.98 (0.45) b	8.16 (0.76) b	7.32 (0.61) b	16.346 ***
Hydroxycinnamic acids (HCAs)										
trans-Caftaric acid	2.22 (0.19) a	2.09 (0.31) a	6.22 (0.87) b	2.12 (0.13) a	55.027 ***	1.02 (0.12)	1.02 (0.08)	1.02 (0.07)	1.01 (0.10)	0.009 (ns)
trans-Coutaric acid	16.79 (1.72)	14.38 (1.65)	13.56 (1.03)	15.37 (1.28)	2.886 (ns)	2.32 (0.65)	2.27 (0.31)	2.32 (0.3)	2.32 (0.13)	0.014 (ns)
Caffeic acid	1.15 (0.04) c	1.00 (0.02) a,b	0.96 (0.03) a	1.13 (0.04) b,c	18.200 ***	nd	nd	nd	nd	-
trans-fertaric acid	0.14 (0.01) a	0.17 (0.01) b	0.22 (0.01) c	0.27 (0.01) c	46.75 ***	nd	nd	nd	nd	-
Ferulic acid	0.43 (0.02) a	0.42 (0.02) a	0.49 (0.02) a	0.60 (0.02) b	10.688 **	nd	nd	nd	nd	-
Total HCAs	20.73 (1.73) b	18.06 (1.68) a	21.45 (1.35) b	19.49 (1.29) a	3.757 (ns)	3.34 (0.66)	3.29 (0.32)	3.34 (0.31)	3.33 (0.16)	0.011 (ns)
Total monomeric phenolics	83.32 (3.06) b	75.88 (3.05) a	76.23 (2.81) a	80.79 (3.33) a,b	4.480 *	67.93 (3.04) c	55.2 (2.67) a	55.41 (2.43) a	56.00 (2.52) a,b	16.741 ***

^a^ Mean values are shown (n = 3). Different letters (a, b, and c) in the same row indicate significant differences (*p* < 0.05). Level of significance: *, **, and *** indicate significance at *p* < 0.05, *p* < 0.01, *p* < 0.001. ^b^ Control wine (C) and wines treated with the PS extracts. WPP: Wine-Purified Polysaccharides, DWRP: Distilled Washing Residues Polysaccharides; CM: Commercial Mannoproteins. ^c^ Nomenclature abbreviation: glc: glucoside; gal: galactoside; glcU: glucuronide. ns: not significant; nd: not detected.

**Table 4:** Concentration of total proanthocyanidins (mg L^−1^) ^a^, % catechin, % epicatechin, % epicatechin terminal subunits and mean degree of polymerization (mDP) of Viura wines treated with the PS extracts and CM after one (T1) and twelve months of bottling (T12).

	T1					T12				
Polymeric Compounds ^b^	C ^c^	WPP ^c^	DWRP ^c^	CM ^c^	F-Value	C ^c^	WPP ^c^	DWRP ^c^	CM ^c^	F-Value
PA	60.47 (3.91)	54.78 (3.33)	55.45 (3.26)	49.33 (2.89)	3.660 (ns)	45.2 (2.95) b	35.74 (1.87) a	34.80 (1.95) a	32.69 (1.83) a	6.986 *
% Cat	10.40 (2.64) b	7.23 (1.23) a	13.07 (1.58) c	11.07 (1.47) b,c	10.695 **	9.5 (2.32) b	7.02 (1.12) a	12.10 (1.32) c	11.02 (1.32) b,c	11.538 **
% Epi	16.13 (2.09) a	21.75 (2.97) b,c	23.59 (2.79) c	18.77 (2.36) a,b	9.774 **	7.02 (0.95) a	9.71 (1.39) b	10.72 (1.24) b	9.17 (1.13) b	10.363 **
% Epi-gal	2.78 (0.31)	2.69 (0.27)	2.69 (0.37)	3.26 (0.59)	2.729 (ns)	1.60 (0.11) a	1.58 (0.10) a	1.49 (0.13) a	1.95 (0.23) b	10.924 **
mDP	2.75 (0.35) c	2.08 (0.23) b	1.61 (0.15) a	1.72 (0.17) a	174.180 ***	3.22 (0.47) c	2.37 (0.24) b	1.62 (0.14) a	1.68 (0.15) a	155.770 ***

^a^ Mean values are shown (n = 3). Different letters (a, b, and c) in the same row indicate significant differences (*p* < 0.05). Level of significance: *, **, and *** indicate significance at *p* < 0.05, *p* < 0.01, *p* < 0.001. ^b^ PA: total proanthocyanidins content (mg L^−1^); Cat: % catechin terminal subunits; Epi: % epicatechin terminal subunits; Epi-gal: % epicatechin gallate terminal subunits; mDP: Mean Degree of Polymerization expressed as the summatory of total subunits divided by the summatory of monomeric Flavan-3-ols. ^c^ Control wine (C) and wines treated with the PS extracts. WPP: Wine-Purified Polysaccharides, DWRP: Distilled Washing Residues Polysaccharides; CM: Commercial Mannoproteins. ns: not significant.

## Data Availability

Not applicable.

## Supplementary Materials

The following supporting information can be downloaded at: https://www.mdpi.com/article/10.3390/molecules28186477/s1. Table S1 Multivariate analysis of variance (MANOVA) of Viura wines compounds (T1 and T12). Percentage of variance attributable (%) of the independent effect of Aging time and PS extract, and the interaction of both (Aging x PS extract). Table S2 Spearman correlation data of Viura wines oenological, phenolic, and sensory analysis parameters.

## Sample Availability

Samples of the compounds are available from the authors.

## Abbreviations

**%** Cat	% catechin terminal subunits
**%** Epi	% epicatechin terminal subunits
**%** Epi-gal	% epicatechin gallate terminal subunits
AG	ArabinoGalactan
AGP	ArabinoGalactan Protein
ANOVA	A one-way analysis of variance
CM	Commercial Mannoproteins
D.O.Ca	Rioja Qualified Denomination of Origin
DAD	Diode Array Detector
DWRP	Distilled Washing Residues Polysaccharides
GP	Glucosyl Polysaccharides
GPA	Generalized Procrustes Analysis
GPC	Gel Permeation Chromatography
HCl	Hydrochloric acid
HGs	HomoGalacturonans
HPLC	High-Performance Liquid Chromatography
IS	Internal Standards
mDP	mean Degree of Polymerization
MeOH	Methanol
MP	MannoProteins
Mw	Molecular weight
OAV	Odor Activity Values
PA	ProAnthocyanidin
PRAG	Polysaccharides Rich in Arabinose and Galactose
PS	PolySaccharides
RG-I	RhamnoGalacturonans type I
RG-II	RhamnoGalacturonans type II
T1	One month aging
T12	Twelve months aging
WPP	Wine-Purified Polysaccharides
