# Supplementary material for: Grape-Derived Polysaccharide Extracts Rich in Rhamnogalacturonans-II as Potential Modulators of White Wine Flavor Compounds
Source: Molecules. 2023 Sep 6;28(18):6477. doi: 10.3390/molecules28186477 (PMC10536722; doi:10.3390/molecules28186477)
Supplement: Supplementary file 1 [file molecules-28-06477-s001.zip › molecules-2532345-supplementary.pdf]

Table S1. Multivariate analysis of variance (MANOVA) of *Viura* wines compounds (T1 and T12). Percentage of variance attributable (%) of the independent effect of Aging time and PS extract, and the interaction of both (Aging x PS extract).

	Aging time			PS extract			Aging x PS extract			
Compounds	F-ratio	*p*-value	% Aging	F-ratio	*p*-value	% PS extract	F-ratio	*p*-value	% Interaction	error (%)
Total Flavonols	110.902	**0.000**	39.42%	49.911	**0.000**	53.23%	1.555	0.239	1.66%	5.69%
Total Flavanols	0.861	0.367	1.54%	9.631	**0.001**	51.80%	3.341	**0.046**	17.97%	28.69%
Total HBAS	3.721	0.072	6.14%	2.465	0.100	12.20%	11.163	**0.000**	55.26%	26.40%
Total HCAS	1364.529	**0.000**	97.36%	3.566	**0.038**	0.76%	3.428	**0.043**	0.73%	1.14%
Total Monomeric Phenolics	278.490	**0.000**	78.80%	17.969	**0.000**	15.25%	1.669	0.213	1.42%	4.53%
Total Polymeric Phenolics	52.825	**0.000**	62.99%	4.219	**0.022**	15.09%	0.795	0.514	2.84%	19.08%
Total Alcohols	1065.930	**0.000**	88.99%	25.397	**0.000**	6.36%	13.219	**0.000**	3.31%	1.34%
Total C6 Alcohols	389.607	**0.000**	40.52%	96.848	**0.000**	30.22%	88.442	**0.000**	27.60%	1.66%
Total Esters	30,753.957	**0.000**	98.22%	106.242	**0.000**	1.02%	74.036	**0.000**	0.71%	0.05%
Total Acetates	1013.624	**0.000**	62.27%	91.874	**0.000**	16.93%	107.493	**0.000**	19.81%	0.98%
Total Acids	1308.872	**0.000**	61.20%	126.883	**0.000**	17.80%	144.338	**0.000**	20.25%	0.75%
Total Volatile Phenols	10,705.579	**0.000**	98.89%	25.125	**0.000**	0.70%	9.681	**0.001**	0.27%	0.15%
Total Terpenes	684.490	**0.000**	66.93%	53.281	**0.000**	15.63%	54.115	**0.000**	15.87%	1.56%

Values in bold showed statistically significant differences in each compound and factor considered (*p*-values < 0.05).

Table S2. Spearman correlation data of *Viura* wines oenological, phenolic, and sensory analysis parameters[a].

		Correlation parameters[b]	Phenolic parameters													
			Total myricetin	Total quercetin	Total kaempferol	Total syringetin	Total Flavonols	Total Flavanols	Gallic acid	Total HCAs[c]	Total monomeric phenolics	PA[c]	%Cat[c]	%Epi[c]	%Epi gal[c]	mDP[c]
Sensory analysis parameters	Sweetness	ϱ Spearman	−0.230	0.218	−0.051	0.368	0.451	−0.058	−0.432	−0.240	0.054	0.214	−0.677*	−0.560	0.010	0.455
		p−value	0.473	0.496	0.876	0.240	0.141	0.857	0.161	0.453	0.866	0.504	0.016	0.058	0.976	0.137
	Smoothness	ϱ Spearman	0.393	−0.218	−0.044	0.172	0.131	0.393	0.218	0.219	0.306	0.480	0.044	0.218	−0.306	0.393
		p−value	0.206	0.495	0.893	0.593	0.685	0.206	0.495	0.495	0.334	0.114	0.893	0.495	0.333	0.206
	Acidity	ϱ Spearman	−0.097	−0.048	0.097	−0.570	−0.628*	−0.290	0.290	0.073	−0.338	−0.676*	0.724**	0.435	0.266	−0.869**
		p−value	0.765	0.882	0.765	0.053	0.029	0.361	0.361	0.823	0.283	0.016	0.008	0.158	0.403	0.000
	Freshness	ϱ Spearman	−0.361	0.206	0.513	−0.199	0.034	−0.176	0.441	−0.021	−0.168	0.231	−0.559	0.303	−0.341	0.378
		p−value	0.248	0.521	0.088	0.536	0.917	0.583	0.151	0.948	0.602	0.470	0.059	0.339	0.278	0.225
	Astringency	ϱ Spearman	−0.821**	0.869**	0.869**	−0.570	00.000	−0.579*	0.241	−0.145	−0.386	−0.531	−0.338	0.193	0.435	−0.145
		p−value	0.001	0.000	0.000	0.053	10.000	0.048	0.450	0.653	0.215	0.076	0.283	0.548	0.157	0.653
	Bitterness	ϱ Spearman	−0.065	0.583*	0.389	−0.255	0.130	00.000	00.000	0.130	0.130	−0.389	0.389	0.130	0.649*	−0.194
		p−value	0.841	0.047	0.212	0.424	0.688	10.000	10.000	0.688	0.688	0.212	0.212	0.688	0.022	0.545
	Body	ϱ Spearman	−0.641*	0.362	0.641*	−0.329	−0.028	−0.418	0.418	−0.140	−0.362	0.028	−0.697*	0.251	−0.251	0.251
		p−value	0.025	0.247	0.025	0.296	0.931	0.176	0.176	0.665	0.247	0.931	0.012	0.432	0.431	0.432
	Duration	ϱ Spearman	−0.065	0.583*	0.389	−0.255	0.130	00.000	00.000	0.130	0.130	−0.389	0.389	0.130	0.649*	−0.194
		p−value	0.841	0.047	0.212	0.424	0.688	10.000	10.000	0.688	0.688	0.212	0.212	0.688	0.022	0.545
	Balance	ϱ Spearman	0.149	−0.596	−0.373	0.284	−0.075	0.075	0.075	−0.075	−0.075	0.522	−0.373	−0.075	−0.672*	0.298
		p−value	0.662	0.053	0.259	0.397	0.828	0.828	0.828	0.827	0.828	0.100	0.259	0.828	0.023	0.373
Oenological parameters	Ethanol degree	ϱ Spearman	0.384	0.384	0.325	0.078	0.355	0.591*	0.473	0.948**	0.591*	0.473	0.562	0.532	0.578*	0.355
		p−value	0.217	0.217	0.302	0.811	0.258	0.043	0.120	0.000	0.043	0.120	0.057	0.075	0.049	0.258
	Titratable acidity	ϱ Spearman	0.384	0.384	0.325	0.078	0.355	0.591*	0.473	0.948**	0.591*	0.473	0.562	0.532	0.578*	0.355
		p−value	0.217	0.217	0.302	0.811	0.258	0.043	0.120	0.000	0.043	0.120	0.057	0.075	0.049	0.258
	Volatile acidity	ϱ Spearman	0.384	0.384	0.325	0.078	0.355	0.591*	0.473	0.948**	0.591*	0.473	0.562	0.532	0.578*	0.355
		p−value	0.217	0.217	0.302	0.811	0.258	0.043	0.120	0.000	0.043	0.120	0.057	0.075	0.049	0.258
	Malic acid	ϱ Spearman	0.384	0.384	0.325	0.078	0.355	0.591*	0.473	0.948**	0.591*	0.473	0.562	0.532	0.578*	0.355
		p−value	0.217	0.217	0.302	0.811	0.258	0.043	0.120	0.000	0.043	0.120	0.057	0.075	0.049	0.258
	pH	ϱ Spearman	0.384	0.384	0.325	0.078	0.355	0.591*	0.473	0.948**	0.591*	0.473	0.562	0.532	0.578*	0.355
		p−value	0.217	0.217	0.302	0.811	0.258	0.043	0.120	0.000	0.043	0.120	0.057	0.075	0.049	0.258
	Free SO2	ϱ Spearman	0.384	0.384	0.325	0.078	0.355	0.591*	0.473	0.948**	0.591*	0.473	0.562	0.532	0.578*	0.355
		p−value	0.217	0.217	0.302	0.811	0.258	0.043	0.120	0.000	0.043	0.120	0.057	0.075	0.049	0.258
	Total SO2	ϱ Spearman	0.384	0.384	0.325	0.078	0.355	0.591*	0.473	0.948**	0.591*	0.473	0.562	0.532	0.578*	0.355
		p−value	0.217	0.217	0.302	0.811	0.258	0.043	0.120	0.000	0.043	0.120	0.057	0.075	0.049	0.258
	Absorbance 420nm	ϱ Spearman	0.384	0.384	0.325	0.078	0.355	0.591*	0.473	0.948**	0.591*	0.473	0.562	0.532	0.578*	0.355
		p−value	0.217	0.217	0.302	0.811	0.258	0.043	0.120	0.000	0.043	0.120	0.057	0.075	0.049	0.258

[a] *Viura* wines oenological, phenolic, and sensory analysis parameters (n = 12).

[b] Correlation parameters. Level of significance * and ** indicates significance at $p < 0.05$ and $p < 0.01$ respectively.

[c] HCAs: Hydroxycinnamic acids; PA: total proanthocyanidins content (mg L^{-1}); Cat: % catechin terminal subunits; Epi: % epicatechin terminal subunits; Epi gal: % epicatechin-gallate terminal subunits; mDP: Mean Degree of Polymerization expressed as the summatory of total subunits divided by the summatory of monomeric Flavan-3-ols.
